# Overcrowding as a risk factor for domestic violence and antisocial behaviour among adolescents in Ejigbo, Lagos, Nigeria

**DOI:** 10.1017/gmh.2016.10

**Published:** 2016-05-03

**Authors:** O. Makinde, K. Björkqvist, K. Österman

**Affiliations:** Peace and Conflict Research & Developmental Psychology, Åbo Akademi University, P.O. Box 311, 65101 Vasa, Finland

**Keywords:** Aggressive behaviour, antisocial behaviour adolescents, domestic violence, Nigeria, Overcrowding

## Abstract

**Background.:**

The objective was to investigate the relationships between overcrowding, domestic violence, and antisocial behaviour in a sample of adolescents in Lagos metropolitan area, Nigeria. Possible gender differences and differences due to religious affiliation concerning domestic violence and antisocial behaviour were also investigated.

**Method.:**

A questionnaire was filled in by 238 Nigerian adolescents, 12–20 years of age; the sample included 122 females (*m* = 15.1 years, s.d. = 2.0) and 116 males (*m* = 15.8 years, s.d. = 2.0). The respondents were from junior and senior secondary schools in Ejigbo and surrounding cities (Isolo, Egbe and Ago-Palace Lagos). Six scales were included: adolescents as victims of adult and sibling aggression, respectively, witnessing of domestic violence, parental negativity towards adolescents, antisocial behaviour among adolescents and poverty in the home. Overcrowding, gender and religious affiliation served as independent variables.

**Results.:**

According to a multivariate analysis of variance with level of poverty as covariate, overcrowding showed significant associations with four of five scales measuring aggressive and antisocial behaviours. Gender and religion were associated with three variables each. However, multiple regression analyses revealed that overcrowding tended to partial out the effects of both gender and religion showing that overcrowding was the most important factor determining negative outcomes.

**Conclusions.:**

The results have implications for housing policies in Nigeria. Moreover, these results may also have implications for research and policy making in other nations and parts of the world.

## Introduction

The world population is growing at an alarming rate. It has been estimated that it will reach 9.7 billion in 2050, and that developing countries, specifically in Africa, will experience most of the growth (UN, 2015). Nigeria is the most populous country in Africa (New World Encyclopedia, 2015). It is also listed second only to India among the countries that are expected to account for the major part of the projected population increase in the world (UN, 2015). The capital city Lagos is extremely crowded; between 2005 and 2010, Lagos was the fastest growing large city in Africa (UN, 2010). It has been projected that by 2020, Lagos will be the biggest city in Africa with over 14 million inhabitants (UN-HABITAT, 2010).

Overcrowding has been defined by the World Health Organization (WHO, 1999) in terms of average floor area per person. A more detailed definition provided by Eurostat (2014) is based on the number of persons per room. It also takes into consideration needs of children and adolescents, a group that is undeniably vulnerable in any overcrowded home or society. According to this definition, a household is considered overcrowded if there is not at least one room per two adolescents of the same gender 12–17 years of age and at least one room per two children under the age of 12.

### Overcrowding in Nigeria

Overcrowding is the norm in Nigeria. This is because Nigerians live in a country where legislation is available but not followed by most of the population. Mostly, legislation is unknown by ordinary citizens due to the high level of illiteracy in the country. Besides, neither the Nigerian constitution nor any other legislation has made provisions about overcrowding among households in Nigeria. Overcrowding in the Nigerian context means that an excessive number of people (children, teenagers and adults) live together in single room apartments, known informally as ‘face-to-face’ apartments, where living conditions are awful and occupants are unable to sleep well due to poor ventilation. There usually is poor hygiene and sanitation and a lack of basic household amenities.

This typical type of building usually occupies an area of 12 × 15 m^2^. It is divided in the middle by a corridor with four rooms on each side. One such building is the home to eight families with an average of six people living in one room of 18 m^2^. One common latrine and a tiny bathroom are situated outside the building. Food is often prepared on the corridor and windows cannot be opened due to security risks. For a detailed description of layout and morphology of this type of housing, see Akinwolemiwa & Gwilliam (2014).

### Lagos metropolitan area

In a study from the area of metropolitan Lagos, it was found that 77% of the randomly selected respondents lived in houses where 5–10 people shared the same sleeping room (Adeyemi *et al*., 2009). Crowding in Lagos is the result of factors such as a low standard of living, migration to find a means of survival and religious and culturally dependent attitudes emanating from having many children to help out with agricultural livelihoods. People in overcrowded communities are commonly also illiterate and do not have information about birth control.

In Lagos, people live in various types of houses, apartments and rooms. The worst and most unthinkable ways of living also exist, such as living in shanties, abandoned or uncompleted buildings, inside or close to rubbish dumps, under bridges and in suburban streets. These people still live, work and go about their normal activities like every other person in the community.

Although the government makes efforts to demolish uninhabitable homes and slums, they continue to spring up in different parts of the city. Despite the fact that the financial ability to relocate people into normal housing is available, the issue of corruption has been an impediment in this matter, so that these uninhabitable so-called ‘homes’ are found in every town, city and village in the country. Moreover, legislation to control immigration is not available and people from all walks of life rush into Lagos city on a daily basis, especially residents from the neighbouring countries such as Benin, Togo and Niger.

### Ejigbo community and its people

The town of Ejigbo is a suburban area southwest of Lagos, known for its dense population. Most people are residents, although Ejigbo also functions as a transit point to other parts of Lagos. As people living in Ejigbo derive their income from outside the town, moving into or out of Ejigbo is a problem, especially during the morning and evening rush hours. A normal morning in the town begins at about 04:30–05:00 am, and the day usually ends between 01 and 02 am. The question that might come to mind is whether the population ever sleeps at all.

The majority of buildings in Ejigbo are usually built without standard architectural layout or planning. This often contributes to flooding and drainage problems. Frequently deficient buildings have to be demolished by the government. Some buildings are so dilapidated that one might suppose they are used to shelter animals. However, that does not deter people from living in them and even renting them out to others.

### Concomitants of overcrowding

A review of the literature shows associations between overcrowding and a variety of both physical and psychological poor health outcomes (Gray, 2001). Although a number of studies have indicated a link between overcrowding and aggressive behaviour, limited research has been done with overcrowding and aggressive behaviours in domestic settings. Gove *et al*. (1979) found that crowding was strongly related to poor social relationships in the home. Another review, specifically concerned with children's health, concluded that in the case of children, crowding is shown to be associated with stress, poor educational outcomes and behavioural problems at school (Evans *et al*., 1998).

The negative impact of overcrowding on the health of city dwellers in Nigeria has also been stressed by Ahianba *et al*. (2008). When associations between crowding and aggressive and antisocial behaviours in children was studied in a sample of Nigerian elementary school boys, it was found that significantly more aggressive boys came from crowded homes as compared with pro-social boys (Ani & Grantham-McGregor, 1998).

### Child abuse and domestic violence in Nigeria

Nigeria has ratified the UN Convention on the Rights of the Child (UN General Assembly, 1989) as well as the African Union Charter on the Rights and the Welfare of the Child (African Commission on Human and Peoples’ Rights, 1990), and the principles have been promulgated into law in 24 out of 36 Nigerian states (UNICEF, 2013). Still, child abuse is usually not recognised in the Nigerian society and little attention is given to it due to other major paediatric problems (Okeahialam, 1984; Wilson-Oyelaran, 1989; Chinawa *et al*., 2014). There is also scarcity of scientific publications regarding the prevalence of child physical abuse in Nigeria, whereas other forms of child abuse, such as sexual, are more frequently reported (Uzodimma *et al*., 2013). Physical abuse of children is also frequent in schools in Nigeria. A number of children have even been reported to have been flogged to death by their teachers (Chianu, 2000).

Domestic violence against women is common in Nigeria. In an article presenting gender-based violence in Nigeria, Ifemeje (2012) described how women and girls often are victims of the utmost cruelty in their own families. The local culture also supports violence against women. In 2013, wife-beating was accepted by 35% of the women in a national survey. This was however a decline from 2008, when it was accepted by 43% (National Population Commission, 2014).

### Research questions

Human development both physiological and psychological is intense during adolescence. The young person goes through a sensitive phase when all kinds of bothers in the form of social norms, rules and customs are questioned and frequently also tested in order for one's own personality to be found. A heightened sense of the need for privacy is often felt in order to successfully transgress into young adulthood. The present study on overcrowding was therefore designed to investigate associations with family-related concomitants in an adolescent population in Nigeria.

The following six variables were included: (a) victimisation from an adult, (b) sibling aggression, (c) witnessing of domestic violence between family members, (d) being a recipient of parental negativity, (e) antisocial behaviour displayed by the adolescents and (f) lack of food or medicine in the home as a measure of poverty.

The following research questions were posed: (i) whether adolescents living in crowded conditions experience more of the above-mentioned problems than do others, (ii) whether a difference between male and female adolescents in respect to these problems exists, or (iii) whether a difference between Christian or Muslim adolescents could be found in this respect, and (iv) whether there is a difference in the strength of the association between overcrowding, gender and religious affiliation independently and jointly with the six variables above.

## Method

### Sample

Five public schools from each of the four cities of Ejigbo, Isolo, Egbe and Ago-Palace were randomly selected for participation in the study. A random selection of junior and senior secondary school classes (1–3) from each school resulted in a total sample of 238 adolescents participating in the study. The age range was 12–20 years. The sample consisted of 122 females (*m* = 15.1 years, s.d. = 2.0) and 116 males (*m* = 15.8 years, s.d. = 2.0). The age difference was not significant. Of the respondents 72.3% (*n* = 172) were Christians and 27.7% (*n* = 66) were Muslims.

A total of 71% (*n* = 169) of the respondents lived in apartments with only one bedroom (crowded conditions), while 29% (*n* = 68) lived in apartments with more than one bedroom.

### Instrument

The data were collected using a paper-and-pencil questionnaire. It included six scales constructed specifically for the study: victimisation from adult aggression, victimisation from sibling aggression, witnessing of domestic violence, parental negativity towards adolescents and antisocial behaviour of adolescents. The items very constructed in focus group discussions in which three persons took part: two of them were experts in psychometrics, and the third an expert in local conditions. Lack of food or medicine in the home served as an indicator of poverty since adolescents seem seldom capable of giving exact information about the family's economic situation, but are likely to know whether there is a lack of food or medicine in the home. Responses were given on a five-point scale (never, seldom, sometimes, often and very often) measuring the degree to which respondents agreed on the statements. Items and reliability scores (Cronbach's *α*) of the scales are presented in Table 1.

**Table 1.** Items and reliability scores of the scales of the study (N = 238)

Scales and items	
Victimisation from adult aggression (*α* = 0.84)	Victimisation from sibling aggression (*α* = 0.91)
Pulled your ears	Pulled your hair
Pulled your hair	Slapped you
Slapped you	Hit you with an object
Hit you with an object	Pinched you
Pinched you	Thrown things at you
Thrown things at you	Twisted your arms
Sleep punishment	Bitten you
Parental negativity towards adolescents (*α* = 0.84)	Witnessing of domestic violence (*α* = 0.82)
Name calling or bullying	Physical fights
Insults	Quarrels
Making and breaking of promises	Thrown things at each other
Constant criticism	Damaged belongings
Intimidation	Twisted each other's arm
Harassment	Stabbed each other
Antisocial behaviour of adolescents (*α* = 0.87)	Poverty (measured as lack of food or medicine) (*α* = 0.79)
Stolen petty things	Food rationing
Used a catapult on a friend or neighbour or someone else	Unavailable or inadequate medication
	Unavailable or inadequate food or drink
Cheated neighbours of their belongings	
Smoked cigarettes	
Unconcentrated at school	
Fighting in school	
Absenteeism from school	

### Procedure

The questionnaires were distributed among junior and senior secondary school students in Ejigbo and the neighbouring towns of Isolo, Egbe and Ago-Palace. A total of 20 public schools, five from each city, participated in the study. The data were collected during a period of 3 months, July–September 2014. Most of the questionnaires were collected from the pupils immediately after completion. Due to the potential sensitivity of the questions, permission was obtained from religious leaders, teachers, parents and school coordinators, prior to the questionnaire being administered.

## Results

### Living conditions of the respondents families

Of the adolescent in the study, 5.3% had a room of their own, 44.9% lived in a room with 2–3 other persons, 36.5% with 3–5 others and 13.3% lived with 6–8 others in the same room. When applying the definition by Eurostat (2014), it was found that 71.1% of the adolescents lived in overcrowded conditions.

The average number of people in the whole sample sleeping in the same room was 3.0 (s.d. = 1.9). Of the female adolescents, 88.8% lived in more than one room apartments, while this was the case for only 54.8% of the males. The difference was significant [*χ*^2^_(1)_ = 33.04, *p* < 0.001]. Families living in one room only apartments scored significantly higher than others on level of poverty [*t*_(229)_ = 8.07, *p* < 0.001]. Of families living in one bedroom apartments, 70.8% were Muslims and 29.2% Christians, and among those who had more than one bedroom, 88.0% were Christians and 12.0% Muslims. Of the families, 24.4% did not have running water, 38.5% cooked their meals in the corridor outside their room(s), and 51.3% of them had access to electricity often or very often, the others less often.

### Relationships between the scales of the study

The six scales of the study were highly inter-correlated. All the correlations were at the 0.001-level. The highest correlation was found between the family's degree of poverty and adolescents perception of parental negativity against them (*r* = 0.68). Poverty was also highly correlated with witnessing of domestic violence (*r* = 0.57). Antisocial behaviours of adolescents were strongly correlated with victimisation from adult aggression at home (*r* = 0.58), and with witnessing of domestic violence between family members (*r* = 0.56). The Pearson correlation coefficients are presented in Table 2.

**Table 2.** Correlations between the scales of the study (N = 238)

	1.	2.	3.	4.	5.
1. Victimisation from adult aggression					
2. Victimisation from sibling aggression	0.54***				
3. Witnessing of domestic violence	0.52***	0.44***			
4. Degree of poverty in the family	0.45***	0.44***	0.57***		
5. Parental negativity towards adolescents	0.47***	0.42***	0.52***	0.68***	
6. Antisocial behaviour of adolescents	0.58***	0.50***	0.56***	0.49***	0.50***

*Note.* *** *p* < 0.001.

### Interaction between gender, religious affiliation and overcrowding

A multivariate analysis of variance (MANOVA) was conducted with gender, religious affiliation and overcrowding as independent variables, and the five scales measuring aggressive and antisocial behaviour as dependent variables. The analysis revealed no significant interaction effects. Accordingly, the independent variables were analysed separately in three different MANOVAs.

### Gender differences

**Fig. 1.**
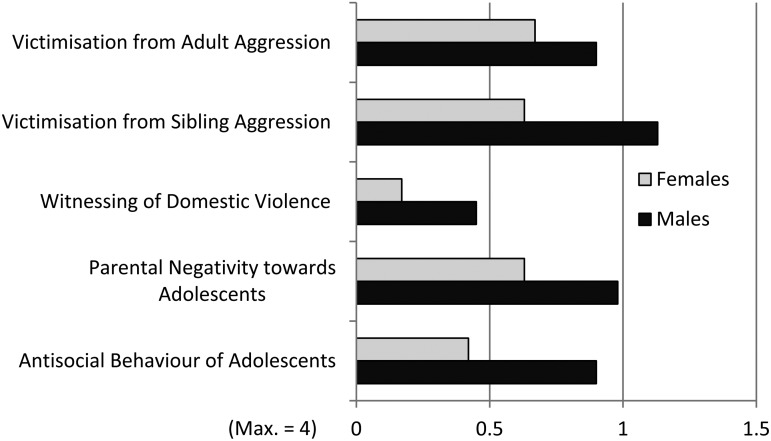
Mean values for female and male adolescents on the five scales (*N* = 238), cf. Table 3.

A MANOVA was performed with gender as independent variable, and five scales as dependent variables. Due to the significant difference in apartment size for females and males in the sample, overcrowding was used as a covariate (Table 3; Fig. 1). It was found that males scored significantly higher than females on victimisation from sibling aggression and on antisocial behaviour of adolescents. A similar tendency was also found for parental negativity towards adolescents. No difference was found between males and females on witnessing of domestic violence and victimisation from adult aggression.

**Table 3.** Results of a MANOVA with five scales as dependent variables, gender as independent variable and overcrowding as covariate (N = 238), cf. Fig. 1

	*F*	df	*p ≤*	*η*_*p*_^2^
Multivariate effect of covariate	12.34	5, 224	0.001	0.216
Multivariate analysis	4.10	5, 224	0.001	0.084
Univariate analyses				
Victimisation from adult aggression	0.00	1, 228	ns	0.000
Victimisation from sibling aggression	6.56	”	0.011	0.028
Witnessing of domestic violence	1.29	”	ns	0.006
Parental negativity towards adolescents	3.78	”	0.053	0.016
Antisocial behaviour of adolescents	10.75	”	0.001	0.045

### Differences related to religious affiliation

**Fig. 2.**
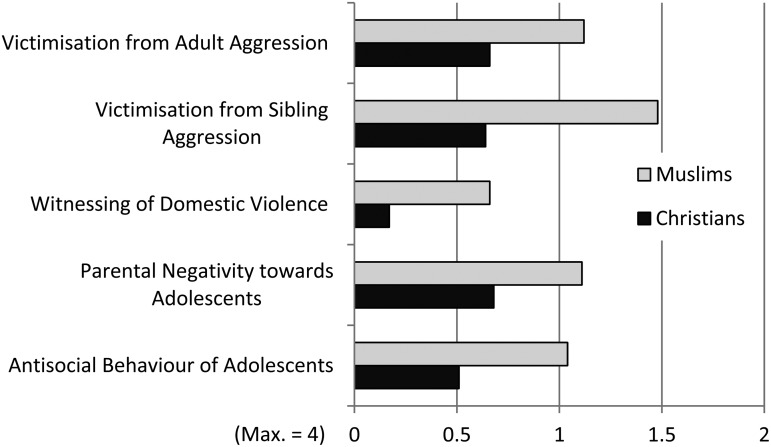
Mean values for Christian and Muslim adolescents on the five scales (*N* = 238), cf. Table 4.

A second MANOVA was performed with five scales as dependent variables and religious affiliation (Christian *v.* Muslim) as an independent variable. Due to the difference in number of rooms between religious groups, overcrowding was kept as covariate (Table 4; Fig. 2). It was found that Muslim adolescents scored significantly higher than Christians on victimisation from sibling aggression, witnessing of domestic violence and antisocial behaviour of adolescents. No differences were found between religious groups on parental negativity towards adolescents and victimisation from adult aggression.

**Table 4.** Results of a MANOVA with five scales as dependent variables, overcrowding as covariate and religion (Christian v. Muslim) as the independent variable (N = 238), cf. Fig. 2

	*F*	df	*p ≤*	*η*_*p*_^2^
Multivariate effect of covariate	6.90	5, 224	0.001	0.133
Multivariate analysis	5.14	5, 224	0.001	0.103
Univariate analyses				
Victimisation from adult aggression	1.77	1, 228	ns	0.008
Victimisation from sibling aggression	22.88	”	0.001	0.091
Witnessing of domestic violence	5.63	”	0.018	0.024
Parental negativity towards adolescents	2.32	”	ns	0.010
Antisocial behaviour of adolescents	4.65	”	0.032	0.020

### Associations between overcrowding and aggressive behaviour

**Fig. 3.**
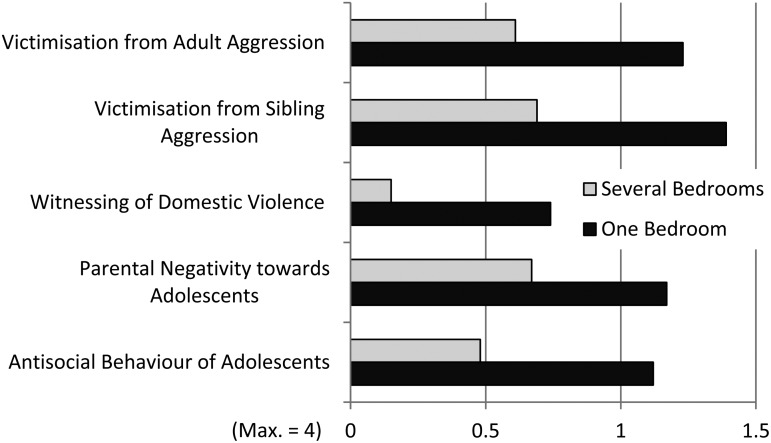
Mean values for adolescents living in one bedroom and more than one bedroom apartments on the five scales (*N* = 238), cf. Table 5.

A third MANOVA was performed with five scales as dependent variables and overcrowding (one bedroom *v.* several bedrooms) as the independent variable. Due to the significant difference regarding economic situation between families living in one or more bedrooms, level of poverty was kept as covariate in the analysis (Table 5; Fig. 3). The results showed that adolescents who lived in apartments with one bedroom only scored significantly higher than adolescents living in more than one bedroom apartments on victimisation from both adult and sibling aggression, witnessing of domestic violence and antisocial behaviour of adolescents. No difference was found for parental negativity towards adolescents.

**Table 5.** Results of a MANOVA with five scales as dependent variables, overcrowding (one bedroom v. several bedrooms) as independent variable and degree of poverty as covariate (N = 231), cf. Fig. 3

	*F*	df	*p ≤*	*η*_*p*_^2^
Multivariate effect of covariate	34.56	5, 224	0.001	0.435
Multivariate analysis	6.61	5, 224	0.001	0.129
Univariate analyses				
Victimisation from adult aggression	10.85	1, 228	0.001	0.045
Victimisation from sibling aggression	12.42	”	0.001	0.052
Witnessing of domestic violence	18.71	”	0.001	0.076
Parental negativity towards adolescents	0.00	”	ns	0.000
Antisocial behaviour of adolescents	16.28	”	0.001	0.067

### A comparison of the effects of overcrowding, gender and religion

As it had been found that all three independent variables in the study, overcrowding, gender and religion, showed significant associations on three or four of the five dependent variables, five linear multiple regression analyses (model Enter), one for each of the dependent variables, were conducted with the three independent variables as predictors (Table 6).

**Table 6.** Results from five multiple linear regression analyses with gender, religion, and overcrowding as predictors, and the five scales of the study as predicted variables

Variables	*β*	*t*	*p ≤*	*R*	*R*^2^	Adj*R*^2^	df	*F*	*p ≤*
Victimisation from adult aggression				0.39	0.15	0.14	3, 227	13.22	0.001
Gender	−0.03	0.41	ns						
Religion	0.11	1.39	ns						
Overcrowding	0.32	4.21	0.001						
Victimisation from sibling aggression				0.48	0.23	0.22	3, 227	22.75	0.001
Gender	0.09	1.40	ns						
Religion	0.32	4.23	0.001						
Overcrowding	0.16	2.25	0.026						
Witnessing of domestic violence									
Gender	0.03	0.52	ns	0.49	0.28	0.23	3, 227	23.52	0.001
Religion	0.16	2.14	0.030						
Overcrowding	0.36	5.00	0.001						
Parental negativity towards adolescents				0.35	0.12	0.11	3, 227	10.39	0.001
Gender	0.11	1.60	ns						
Religion	0.08	1.04	ns.						
Overcrowding	0.23	2.94	0.004						
Antisocial behaviour of adolescents				0.46	0.23	0.22	3, 227	22.05	0.001
Gender	0.18	2.80	0.006						
Religion	0.10	1.35	ns						
Overcrowding	0.30	4.09	0.001						

The gender of the respondents significantly predicted one variable: antisocial behaviour, whereas religious affiliation predicted two: victimisation from sibling aggression and witnessing of domestic violence between family members. Overcrowding on the other hand, was found to be a significant predictor of all five variables in the study. It can thus be concluded that overcrowding appears to be a prominent source for different problems in the daily life of adolescents in the Lagos area.

## Discussion

The results showed that adolescents who lived in crowded homes, measured in terms of number of bedrooms, scored significantly higher than others on being victimised from both adult and sibling aggression, and also on witnessing of domestic violence between family members. Furthermore, they also reported more antisocial behaviour problems. They did however not perceive themselves as more subjected to parental negativity than other adolescents. Since families living in one room apartments displayed a higher level of poverty, poverty was kept in as a covariate in the analyses.

It was found that adolescent males scored higher than adolescent females on victimisation from sibling aggression. They also tended to perceive themselves as targets of more parental negativity than females. Adolescent males also scored significantly higher on antisocial behaviour. Degree of crowding was kept as a covariate in these analyses.

Since the majority of the families in the study living in only one bedroom apartments were Muslims, these families were likely to experience crowding to a significantly higher degree than others. Overcrowding was accordingly kept as a covariate when analyses of differences due to religion were made. It was shown that there were no significant differences between religious groups on victimisation from adult aggression or on parental negativity towards adolescents. Muslim adolescents reported higher scores on being victimised from sibling aggression, witnessing of domestic violence and antisocial behaviour.

According to multiple regression analyses, overcrowding was found to be a significant predictor of all five outcome variables in the study, whereas gender predicted only one, and religion two of the variables.

Overcrowding thus appears to be an influential factor in domestic life in Lagos predicting victimisation of adolescents from both adult and sibling aggression, as well as witnessing of domestic aggression between family members, being themselves recipients of parental negativity, and having a significantly higher level of antisocial problems.

Poverty and overcrowding are strongly interlinked phenomena and difficult to separate in a meaningful way. It should be noted that the level of poverty was in this study kept as a covariate. That means that the effect of poverty as such was partialled out and that a clear negative outcome due to overcrowding could still be observed.

### Limitations of the study

Since the study was not longitudinal, overcrowding cannot unequivocally be shown to be the cause of these negative outcomes especially as only associations between variables were observed. Generalisations of the results to other parts of Nigeria should be made with caution taking special characteristics of that area into consideration.

### Implications of the study

Housing policies in Nigeria and other densely populated areas in the world would benefit from taking into account psychosocial concomitants of domestic overcrowding. Special focus should be placed on the needs of adolescents. The complex relationship between overcrowding and domestic aggression is still to be investigated in more depth. Possible mediating variables connected to overcrowding should also be investigated. Such mediating variables could be stress, heat, noise and the absence of privacy in large families inhabiting one small overcrowded room. Further research on both psychological and behavioural concomitants of overcrowding among all age groups is also essential.
